# Coenzyme Q10 in acute influenza

**DOI:** 10.1111/irv.12608

**Published:** 2018-09-30

**Authors:** Maureen Chase, Michael N. Cocchi, Xiaowen Liu, Lars W. Andersen, Mathias J. Holmberg, Michael W. Donnino

**Affiliations:** ^1^ Department of Emergency Medicine Beth Israel Deaconess Medical Center Boston Massachusetts; ^2^ Division of Critical Care Department of Anesthesia Critical Care Beth Israel Deaconess Medical Center Boston Massachusetts; ^3^ Research Center for Emergency Medicine Aarhus University Hospital Aarhus Denmark; ^4^ Division of Pulmonary Critical Care & Sleep Medicine Department of Medicine Beth Israel Deaconess Medical Center Boston Massachusetts

## Abstract

**Objectives:**

The goal of this investigation was to determine if acute influenza infection is associated with depletion of CoQ10 compared to healthy controls and to determine any associations between CoQ10 levels and illness severity and inflammatory biomarkers.

**Patients and Methods:**

We analyzed serum CoQ10 concentrations of patients with acute influenza enrolled in a randomized clinical trial prior to study drug administration. Patients were enrolled at a single urban tertiary care center over 3 influenza seasons (December 27, 2013 to March 31, 2016). Wilcoxon rank sum test was used to compare CoQ10 levels between influenza patients and healthy controls. Correlations with inflammatory biomarkers and severity of illness were assessed using Spearman correlation coefficient.

**Results:**

We analyzed CoQ10 levels from 50 patients with influenza and 29 controls. Overall, patients with acute influenza had lower levels of CoQ10 (.53 μg/mL, IQR .37‐.75 vs .72, IQR .58‐.90, *P* = .004). Significantly more patients in the influenza group had low CoQ10 levels (<.5 μg/mL) compared to controls (48% vs 7%, *P* < .001). Among influenza patients, there were significant but weak correlations between CoQ10 levels and IL‐2 (*r* = −.30, *P *= .04), TNF‐alpha (*r* = −.35, *P* = .01) and VEGF (*r* = .38, *P* = .007), but no correlation with IL‐6, IL‐10, VCAM or influenza severity of illness score (all *P* > .05).

**Conclusions:**

We found that CoQ10 levels were significantly lower in patients with acute influenza infection and that these levels had significant although weak correlations with several inflammatory biomarkers.

## 1 INTRODUCTION

Seasonal and pandemic influenza remains a wide‐scale public health problem despite aggressive influenza vaccination programs in many countries. The World Health Organization (WHO) estimates 3‐5 million cases of severe influenza illness worldwide and attributes 250 000‐500 000 deaths to seasonal influenza outbreaks each year.^1^ Over the past five influenza seasons, the Centers for Disease Control (CDC) attributes anywhere from 12 000‐56 000 deaths each year to influenza across all age ranges in the United States.^2^ However, this may be an underestimation as we know that beyond the direct mortality effects, mortality rates in other major diseases also peak when influenza is circulating.^3^, ^4^ While the majority of influenza‐attributable deaths occur in patients > 65 years old, the 2009 H1N1 pandemic resulted in a trend toward severe illness and death in younger, otherwise healthy patients.^5^, ^6^, ^7^, ^8^

As influenza therapies are currently limited to antiviral agents that may have variable effect on outcomes, it is important to investigate host factors that may be affected by acute influenza infection and could hold diagnostic, prognostic, or therapeutic potential.^9^ Coenzyme Q (CoQ10) or ubiquinone is an integral component of the mitochondrial respiratory chain and antioxidant. Levels of CoQ10 can become depleted in both acute and chronic illness leading to decreased cellular energy production and increased free radicals which can, in turn, further damage mitochondria.^10^ Prior investigations have demonstrated that CoQ10 levels are decreased in a broad range of critically ill patients,^11^ and previous investigations from our group have found low CoQ10 levels in patients with septic shock and cardiac arrest.^12^, ^13^In patients with septic shock, low CoQ10 levels were related to inflammatory cytokines and vascular endothelial markers.^13^ In patients who initially survived a cardiac arrest, low CoQ10 levels were associated with both mortality and poor neurologic outcomes.^12^

To our knowledge, CoQ10 has not been studied in adult patients with influenza. We hypothesized that acute influenza infection may result in a depletion of CoQ10 levels. We additionally sought to identify associations between CoQ10 levels and influenza infection severity and inflammatory biomarkers.

## 2 METHODS

### 2.1 Study design

This was an observational sub‐study of an ongoing single center, randomized, double‐blind, placebo‐controlled trial comparing atorvastatin to placebo in patients presenting with confirmed influenza (ClinicalTrials.gov Identifier: NCT02056340). In brief, patients were randomized to receive either atorvastatin 40 mg or matching placebo capsule orally daily for 5 days or to a maximum of seven days while hospitalized. Patients were also asked to rate their influenza symptoms daily for 10 days generating a composite influenza severity of illness score based on five major symptoms (fever, cough, sore throat, headache, and myalgias) ranked from 0 to 3 (none, mild, moderate, and severe) for a daily score ranging from 0 to 15. The primary endpoint in the trial is the change in inflammatory biomarkers from time zero to 72 hours. As part of an exploratory analysis, we analyzed baseline CoQ10 levels in patients enrolled in the trial prior to study drug administration. The study was approved by the Institutional Review Board.

### 2.2 Study setting and patient population

The study was conducted at Beth Israel Deaconess Medical Center which is an urban tertiary care teaching hospital affiliated with Harvard Medical School in Boston, MA. All patients presenting to the medical center with an influenza‐like illness were screened for enrollment between December 2013 and May 2016. Study eligible patients were adults >18 years with acute influenza confirmed by either a bedside rapid antigen test (RAT) or a documented hospital laboratory test (direct fluorescence antibody or polymerase chain reaction). Primary screening occurred in the Emergency Department but hospitalized inpatients with a positive flu test were also screened. Patients were excluded if they had concomitant or recent (within 30 days) statin medication use, were pregnant or actively breastfeeding, had cirrhosis or acute liver dysfunction with alanine and aspartate aminotransferase (ALT/AST) > 240 international units/L, creatinine phosphokinase (CPK) 3x above the upper limit of normal, had an allergy or previous intolerance to statin medications, were unable to tolerate oral or nasogastric medications, had a do not resuscitate or comfort measures only designation, were a member of a protected population, or were otherwise unable to provide written informed consent. Patients were further excluded if they were taking any medications contraindicated with concomitant atorvastatin administration, including cyclosporine, HIV protease inhibitors, gemfibrozil, niacin, azole antifungals, clarithromycin, and colchicine. As this was an exploratory and secondary analysis, we included consecutive influenza subjects who had in excess of four serum samples available for analysis. For comparison to CoQ10 levels in acute influenza patients, control measurements were drawn from a repository of healthy volunteers representing both hospital employees and Emergency Department patients with minor complaints such as mild musculoskeletal injuries (sprains), superficial lacerations, and medication refills. A standardized questionnaire was employed to screen controls regarding basic demographic information and past medical history. For the exploratory aims in the larger trial, we initially calculated that a sample size of 50 patients in the control and influenza groups would allow us to detect an effect size of 0.5 with 80% power (two‐sided alpha 0.1). We reduced the number of control subjects as we had previously measured CoQ10 levels in a similar population of healthy controls and found lower variance than expected and values consistent with previously reported normal ranges.^13^, ^14^

### 2.3 CoQ10 measurements

Serum CoQ10 levels were measured by high‐pressure liquid chromatography (HPLC) at the Laboratory of Metabolic and Mitochondrial Diseases at Columbia University Medical Center in New York. This analysis has been previously described in detail.^13^ Normal values for CoQ10 as measured by HPLC with the electrochemical detection system (ESA Coulochem II, ESA, Inc, Chelmsford, MA) are 0.8 ± 0.2 µg/mL. Values lower than 0.5 µg/mL are regarded as low.

### 2.4 Biomarker analysis

Influenza patient plasma samples were analyzed for multiple vascular endothelial and inflammatory markers (VEGF, VCAM‐1, IL‐2, IL‐6, IL‐10, and TNF‐alpha) using customized Meso Scale Discovery Human Multiplex Panel (Rockville, MD, USA) at Beth Israel Deaconess Medical Center. All samples were measured in duplicate with the inter‐assay coefficients of variability ranging from 2.2 to 5.8%. VCAM‐1 is reported in log‐transformed μg/mL and the rest of the markers in log‐transformed pg/mL.

### 2.5 Statistical analysis

Patient demographics and clinical characteristics are reported using descriptive statistics. Continuous data are reported as means with standard deviations or medians with 1st and 3rd quartiles depending on normality of the data, and categorical data are reported as counts with relative frequencies. The Wilcoxon rank‐sum test was used to compare CoQ10 levels between groups. Fisher's exact test was used to compare the proportion of influenza patients and healthy controls with low CoQ10 levels. The relationship between CoQ10 levels and inflammatory biomarkers was assessed using the Spearman correlation coefficient as the data were not normally distributed. At the time of processing, one sample was identified as having insufficient volume for the biomarker assay, resulting in 49 patients with inflammatory biomarker correlation analysis performed. Statistical analyses were performed using SAS software, version 9.4 (SAS Institute Inc., Cary, NC, USA).

## 3 RESULTS

**Figure 1:**
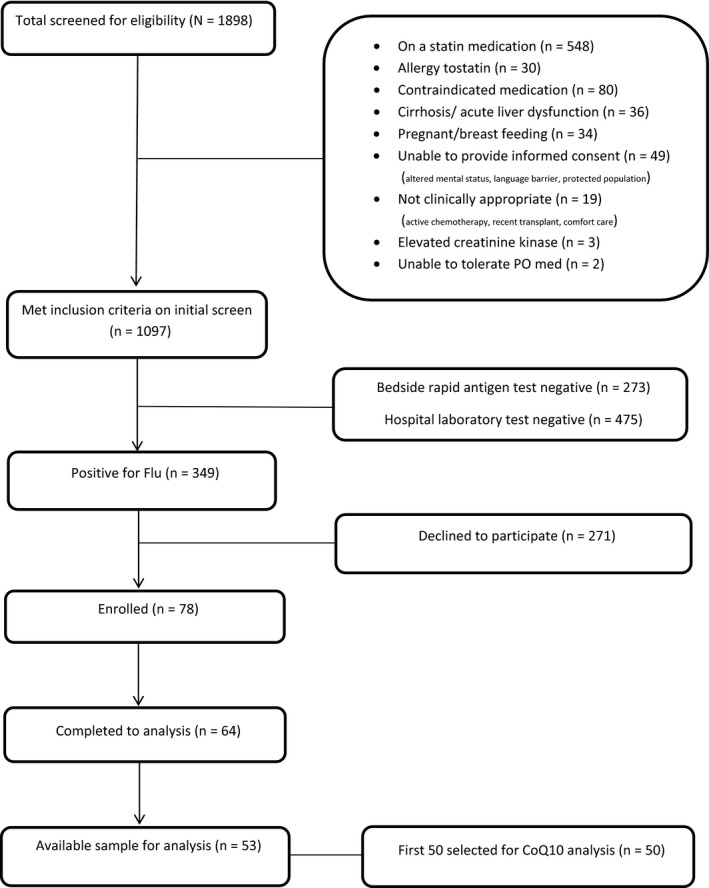
Screening and Enrollment Flowchart

We analyzed CoQ10 levels from the first 50 subjects enrolled in the original trial with available samples (Figure 1). The median age was 40 (IQR 27‐54) years, 48% were female, and 34% were Black/African American. Forty‐two percent had no preexisting medical history. We compared CoQ10 levels to 29 healthy controls with a median age of 26 (IQR 24‐35) years. Additional influenza patient and control subject characteristics are described in Table 1.

**Table 1:** Study subject demographics^a^

	Influenza patients (n = 50)	Controls (n = 29)
Demographics		
Age (years) (median, IQR)	40, 27‐54	26, 24‐35
Sex (female)	24 (48)	24 (83)
Race		
Black/ African American	17 (34)	0 (0)
Caucasian, non‐Hispanic	24 (48)	26 (90)
Caucasian, Hispanic	8 (16)	3 (10)
Asian	1 (2)	0 (0)
Day 1 Influenza symptom score (mean, SD)	8.3 ± 3.3	Not applicable
CoQ10 level (µg/mL) (median, IQR)	0.53, 0.37‐0.75	0.72, 0.58‐0.90
Low CoQ10 (<0.5 µg/mL)	24 (48)	2 (7)
Past medical history		
None	21 (42)	25 (86)
Hypertension	9 (18)	3 (10)
Diabetes	2 (4)	0 (0)
Asthma	9 (18)	0 (0)
Cancer	3 (6)	0 (0)
COPD	4 (8)	0 (0)
Stroke	1 (2)	0 (0)
Obesity	3 (6)	0 (0)
Tobacco use	3 (6)	Not recorded
Immunized for influenza in current season	15 (31)	Not applicable
Initial disposition		
Discharged from ED	34 (68)	Not applicable
ED observation	6 (12)	Not applicable
Floor admission	7 (14)	Not applicable
ICU admission	3 (6)	Not applicable

a Categorical variables are presented as count (frequency) and continuous variables as median (quartiles) or means ([SD] standard deviation).

**Figure 2:**
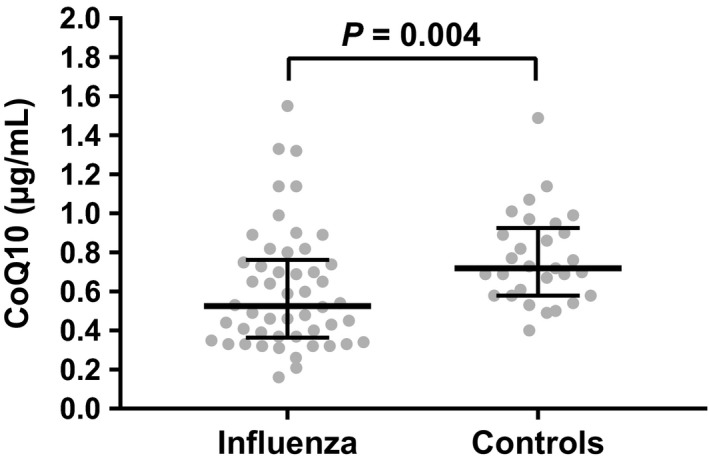
Box Plot of CoQ10 levels between patients with influenza and controls

**Figure 3:**
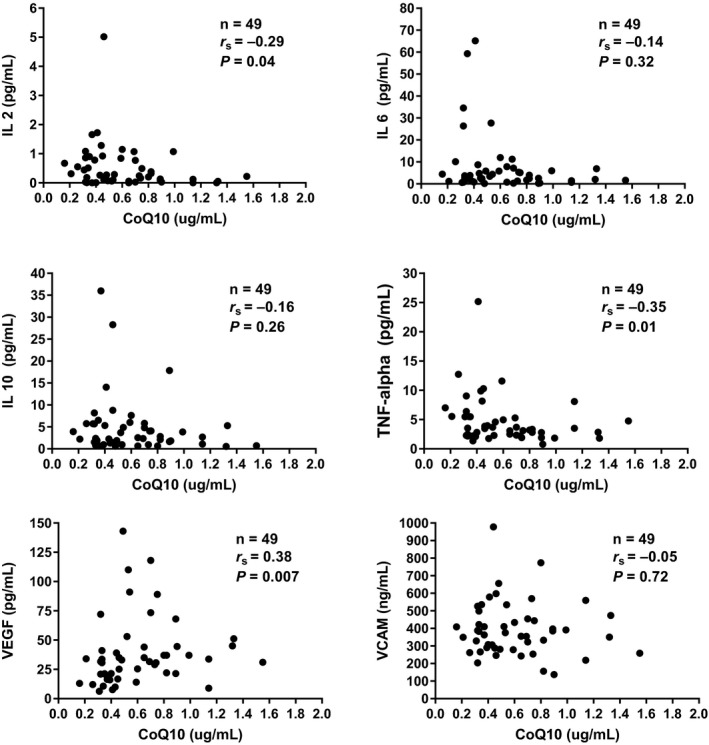
Correlations between CoQ10 and individual biomarkers

Overall, influenza patients had significantly lower levels of CoQ10 as compared to controls (median 0.53, IQR 0.37‐0.75 vs 0.72, IQR 0.58‐0.90 µg/mL, *P* = 0.004) (Figure 2). Twenty‐four patients in the influenza group (48%) had low CoQ10 levels (<0.5 µg/mL) as compared to 7% of control patients (*P* < 0.001). Exactly 50% of influenza patients with low CoQ10 levels (n = 12) had no comorbid medical conditions. When examining potential correlations between CoQ10 levels, clinical factors, and inflammatory biomarkers, we found no correlation between CoQ10 levels and patient‐reported influenza symptom severity score at time of enrollment (*r* = −0.06, *P* = 0.69) or with plasma IL‐6 levels (*r* = −0.14, *P* = 0.32), VCAM (*r* = −0.05, *P* = 0.72) or IL‐10 (*r* = −0.16, *P* = 0.26). We did find significant correlations between CoQ10 levels and other inflammatory biomarkers as follows: IL‐2 (*r* = −0.30, *P* = 0.04), TNF‐alpha (*r* = −0.35, *P* = 0.01), and VEGF (*r* = 0.38, *P* = 0.007). Of note, VEGF was the only marker with a positive correlation with CoQ10 levels. These results are detailed in Figure 3. When we restricted this analysis to the subset of 24 patients who had low (<0.5 µg/mL) CoQ10 levels, we did not find any significant correlations with inflammatory biomarkers.

## 4 DISCUSSION

In the present investigation, we hypothesized that CoQ10 levels would be decreased in the setting of acute influenza infection and we found that nearly 50% of our influenza study subjects had low CoQ10 levels at time of study enrollment compared to only 7% of healthy controls. We further found that CoQ10 levels in our influenza subjects were inversely correlated with certain inflammatory markers, but not others.

We are only aware of one prior study on CoQ10 levels in influenza infection in a pediatric population which demonstrated that CoQ10 levels were significantly lower in children infected with pandemic H1N1 influenza compared to both controls and to those infected with seasonal influenza. However, the authors did not find any significant difference between CoQ10 levels in patients with seasonal influenza and controls. In fact, CoQ10 levels in patients with seasonal influenza were actually higher than in controls in their population.^15^ The reasons for the disparate findings between control subjects and those infected with seasonal influenza between this study and our investigation remain unclear but may be related to the age difference between the populations studied (pediatric vs adult) and the potential differences in response to oxidative stress with age. The authors of the pediatric study also looked at total antioxidant capacity and found that it was decreased in both pandemic and seasonal influenza compared to controls. While we did not specifically evaluate total antioxidant capacity in our investigation, we do know that influenza infection causes excessive accumulation of reactive oxygen species which promote the inflammatory response via signaling pathways, ultimately leading to cell injury and contributing to overall influenza‐induced pathogenesis.^16^, ^17^ Beyond this, Hennet et al.^18^ found that key cellular antioxidants which would help to neutralize reactive oxygen species were depleted early in influenza infection; in a mouse model, they observed a reduction in the total concentrations of the hepatic antioxidants glutathione and vitamins C and E. The combined effect of influenza‐induced oxidative stress and depleted antioxidant concentrations has lead several investigations to study antioxidants as potential therapeutic strategies against influenza pathogenesis with encouraging findings.^19^, ^20^, ^21^ Whether or not CoQ10 is such an adjuvant therapy has yet to be determined but the current study is the first, to our knowledge, to suggest that CoQ10 might be similarly affected in acute influenza infection.

We also know that a dysregulated inflammatory response elicited by the influenza virus, the so‐called “cytokine storm,” contributes significantly to the infection‐associated morbidity and mortality.^22^, ^23^, ^24^ Specifically, the inflammatory cytokines IL‐6 and TNF‐alpha have been most heavily implicated in the pathogenicity of influenza infection; multiple human studies have demonstrated their association with progression of disease, severe illness and death, leading some investigators to conclude that immunomodulatory therapies are necessary to stop the excessive cytokine activation.^25^, ^26^, ^27^, ^28^A recent meta‐analysis of clinical trials on CoQ10 administration supports the role of CoQ10 as a moderator of the inflammatory response.^29^ Specifically, in chronic conditions related to the overproduction and over‐activity of pro‐inflammatory cytokines, CoQ10 supplementation resulted in the attenuation of inflammatory markers and prevented the activation of inflammatory signaling pathways. For example, among patients with rheumatoid arthritis, investigators found that TNF‐alpha was suppressed after two months of CoQ10 supplementation as compared to placebo.^30^ In another trial of multiple sclerosis patients treated with CoQ10, subjects who received CoQ10 therapy had both lower levels of TNF‐alpha and IL‐6 compared to placebo after 12 weeks.^31^ Given the potential anti‐oxidative and anti‐inflammatory capacity of CoQ10, we expected our finding of low levels of CoQ10 to be associated with an increase in inflammatory markers. In this investigation, we found a statistically significant inverse weak correlation between CoQ10 levels and IL‐2 and TNF‐alpha. While the role of elevated TNF‐alpha in influenza pathogenesis has been well‐studied, the association between an overactive IL‐2 response and morbidity in influenza infection is less clear. In fact, several studies have demonstrated a restoration of the T cell mediated response to either influenza infection or vaccination with the addition of IL‐2 in vitro in older subjects in whom this type of immunity has waned.^32^, ^33^ While our findings of an inverse relationship between CoQ10 and TNF‐alpha and IL‐2 are associative, a previous laboratory investigation of CoQ10 in peripheral blood mononuclear cells (PBMCs) suggests a potential causal relationship; Bessler et al. reported decreased TNF‐alpha and IL‐2 secretion in PBMC incubated with varying doses of CoQ10 for 24 hours.^34^ In patients with septic shock, Donnino et al.^13^ also found a significant association between CoQ10 and IL‐2 and TNF‐alpha.

In this study, we also found a weak positive correlation between VEGF and CoQ10. VEGF is known to induce vascular permeability, and prior studies have demonstrated that VEGF is elevated and correlated to severe disease, shock, and mortality in patients with sepsis.^35^, ^36^, ^37^ We are only aware of two animal studies of VEGF in influenza. In one, the authors attempted to ameliorate the vascular hyperpermeability in lung tissue resulting from cytokine response to influenza infection by administering a soluble ligand that acts as an inhibitor of VEGF‐induced vascular permeability.^38^ They found both reduced lung hyperpermeability and reduced mortality in the treated mice with no effect on either cytokine levels or viral load. In the second, Jang and colleagues studied the brains of mice infected with influenza and found that VEGF was not induced during the initial phase of infection but did rise later in the infectious course.^39^ Neither of these animal studies puts our findings into context but may provide a basis for future investigation into the relationship between CoQ10 and VEGF.

While IL‐6 has been reported to correspond with the magnitude of viral replication and disease severity in influenza infection as referenced above, we did not find a correlation with this specific cytokine in our cohort. Previous investigations from our group have found associations between CoQ10 levels and IL‐6 in acute critical illness. Donnino et al., reported a significant relationship between plasma CoQ10 levels and IL‐6 in patients with septic shock, and Cocchi et al. found a significant inverse correlation between CoQ10 levels and IL‐6 in a post‐cardiac arrest cohort.^12^, ^13^ Compared to these prior studies, our findings of no correlation between CoQ10 levels and IL‐6 were unexpected and remain largely unexplained. It is possible that the cohort in these previous studies reflected a more severely ill population at the time of the measurements compared to the current study, which may, in part, explain the observed differences. Further understanding of the patterns of injury and host pro‐inflammatory responses may reveal potential therapeutic targets in these conditions.

We also did not find any correlation between CoQ10 levels and clinical severity of illness as reported by the patient. This finding is consistent with Coppadoro et al.^11^ who found low CoQ10 levels in a heterogeneous population of critically ill patients but found no correlation between these levels and severity of illness scores or mortality.

In the ongoing primary trial, patients with acute influenza are randomized to treatment with either atorvastatin or placebo daily. Statin medications target 3‐hydroxy‐3‐methylglutaryl coenzyme A reductase (HMG Co‐A reductase), which is a key enzyme in cholesterol synthesis and also plays a key role in the biosynthesis of CoQ10.^40^, ^41^ Our results demonstrate that patients with acute influenza infection have lower circulating CoQ10 levels at the time that they were enrolled in the trial. While the present investigation is exploratory, at the conclusion of the primary trial, we will be able to compare CoQ10 levels between controls and those receiving atorvastatin to assess whether there is any effect on CoQ10 levels during the brief treatment window, thereby providing additional data on this topic. However, Donnino et al.^13^ evaluated the CoQ10 levels of 14 septic shock patients treated with simvastatin therapy or placebo did not find a significant change in mean CoQ10 levels between the treatment and control groups after 72 hours of therapy.

This study was an exploratory analysis of a larger trial. As such, it is not without limitations. Most significantly, our control and influenza subjects had differences in age, sex, race, and comorbid conditions. We cannot therefore draw any definite conclusions about the effect of acute influenza infection on CoQ10 levels. Our internal controls are in keeping with published normal ranges of CoQ10, and we do find the proportion of patients with low CoQ10 in our influenza cohort, particularly among those with no reported comorbid conditions, relative to controls intriguing and worthy of further study.

In conclusion, in this study, we found that patients with acute influenza had significantly lower CoQ10 levels than healthy controls at time of enrollment. CoQ10 levels in influenza patients also had a significant although weak correlation with several inflammatory biomarkers, though not with others which have been implicated in influenza pathogenesis (IL‐6). The patterns of correlation between CoQ10 and the inflammatory markers studied may reflect the pattern of injury and response in acute influenza. Further investigation is warranted to determine if CoQ10 could represent a key therapeutic target in influenza infection.

## CONFLICT OF INTEREST

Dr. Donnino received an investigator‐initiated grant from Kaneka to study ubiquinol (reduced Coenzyme Q10) in patients with sepsis and later in patients with cardiac arrest. Dr. Donnino does not consult for or have any relationship with Kaneka outside of the investigator‐initiated research.
